# 4-(Diphenyl­amino)­benzaldehyde 4-phenyl­thio­semicarbazone

**DOI:** 10.1107/S160053681203053X

**Published:** 2012-07-10

**Authors:** Rafael Mendoza-Meroño, Laura Menéndez-Taboada, Santiago García-Granda

**Affiliations:** a Departamento de Química Física y Analítica, Facultad de Química, Universidad de Oviedo – CINN, C/ Julián Clavería, 8, 33006 Oviedo, Spain

## Abstract

The title mol­ecule, C_26_H_22_N_4_S, is composed of three main parts, *viz.* a triphenyl­amine group is connected to a phenyl ring by a thio­semicarbazone moiety. The C= N double bond has an *E* conformation. The crystal packing is dominated by strong hydrogen bonds through the thio­semicarbazone moiety, with pairs of N—H⋯S hydrogen bonds linking the mol­ecules to form inversion dimers with an *R*_2_^2^(8) ring motif. An intra­molecular N—H⋯N hydrogen bond is also present, generating an *S*(5) ring motif. Although the structure contains four phenyl rings, π–π stacking inter­actions are not formed between them, probably due to the conformation adopted by the triphenyl­amine group. However, a weak π–π stacking inter­action is observed between the phenyl ring and the delocalized thio­semicarbazone moiety.

## Related literature

For related compounds and their biological activity, see: Gupta *et al.* (2007 ▶); Lee *et al.* (2010 ▶); Odenike *et al.* (2008 ▶). For hydrogen-bond motifs, see Bernstein *et al.* (1995 ▶). For a description of the Cambridge Structural Database, see: Allen (2002 ▶).
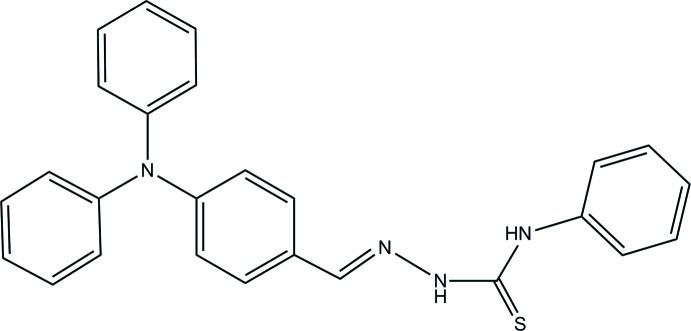


## Experimental

### 

#### Crystal data

C_26_H_22_N_4_S
*M* *_r_* = 422.55
Monoclinic, 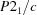

*a* = 13.6069 (3) Å
*b* = 15.2763 (3) Å
*c* = 11.2778 (2) Å
β = 104.094 (2)°
*V* = 2273.67 (8) Å^3^
*Z* = 4
Cu *K*α radiation
μ = 1.41 mm^−1^
*T* = 285 K
0.17 × 0.09 × 0.05 mm

#### Data collection

Oxford Xcalibur diffractometer with Onyx Nova detector
Absorption correction: multi-scan (*CrysAlis PRO*; Oxford Diffraction, 2010 ▶) *T*_min_ = 0.726, *T*_max_ = 1.000
26370 measured reflections
4623 independent reflections
3535 reflections with *I* > 2σ(*I*)
*R*_int_ = 0.067

#### Refinement

*R*[*F* ^2^ > 2σ(*F* ^2^)] = 0.049
*wR*(*F* ^2^) = 0.149
*S* = 1.07
4623 reflections
369 parameters
All H-atom parameters refined
Δρ_max_ = 0.20 e Å^−3^
Δρ_min_ = −0.29 e Å^−3^

### 

Data collection: *CrysAlis CCD* (Oxford Diffraction, 2010 ▶); cell refinement: *CrysAlis RED* (Oxford Diffraction, 2010 ▶); data reduction: *CrysAlis RED*; program(s) used to solve structure: *SIR08* (Burla *et al.*, 2007 ▶); program(s) used to refine structure: *SHELXL97* (Sheldrick, 2008 ▶); molecular graphics: *ORTEP-3 for Windows* (Farrugia, 1997 ▶) and *Mercury* (Macrae *et al.*, 2008 ▶); software used to prepare material for publication: *WinGX* (Farrugia, 1999 ▶), *PLATON* (Spek, 2009 ▶), *PARST95* (Nardelli, 1995 ▶) and *publCIF* (Westrip, 2010 ▶).

## Supplementary Material

Crystal structure: contains datablock(s) global, I. DOI: 10.1107/S160053681203053X/lr2069sup1.cif

Structure factors: contains datablock(s) I. DOI: 10.1107/S160053681203053X/lr2069Isup2.hkl

Supplementary material file. DOI: 10.1107/S160053681203053X/lr2069Isup3.cml

Additional supplementary materials:  crystallographic information; 3D view; checkCIF report

## Figures and Tables

**Table 1:** Hydrogen-bond geometry (Å, °)

*D*—H⋯*A*	*D*—H	H⋯*A*	*D*⋯*A*	*D*—H⋯*A*
N4—H28⋯N2	0.88 (3)	2.19 (3)	2.629 (2)	110 (2)
N3—H27⋯S1^i^	0.98 (2)	2.36 (3)	3.318 (2)	169 (2)

Symmetry code: (i) 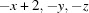.

**Fig. 1.**
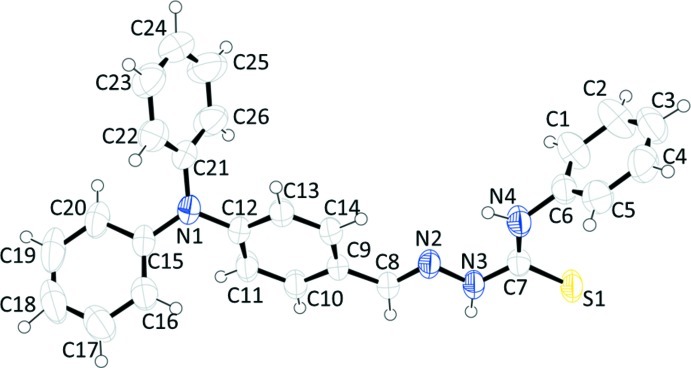
A view of the molecular structure of the title molecule. Displacement ellipsoids are drawn at the 50% probability level.

**Fig. 2.**
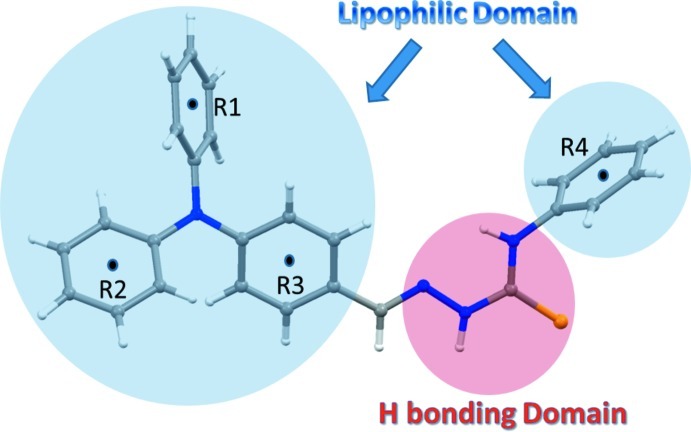
The three main parts of the molecular structure, divided into apolar groups (R1, R2, R3, R4) and H-bonding domain.

**Fig. 3.**
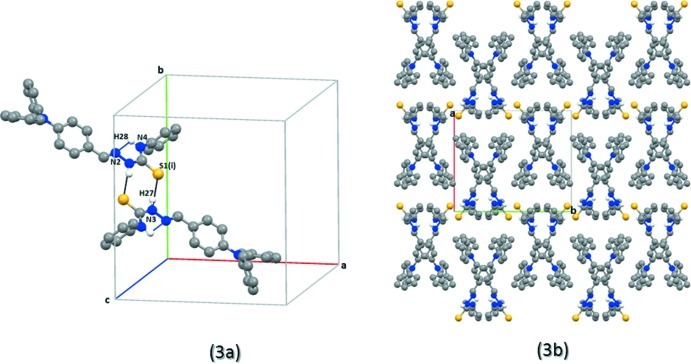
(a) Intermolecular and intramolecular hydrogen bonds, atoms not involved in hydrogen bonding have been omitted for clarity. (b) Packing diagram viewed down the c axis.

**Fig. 4.**
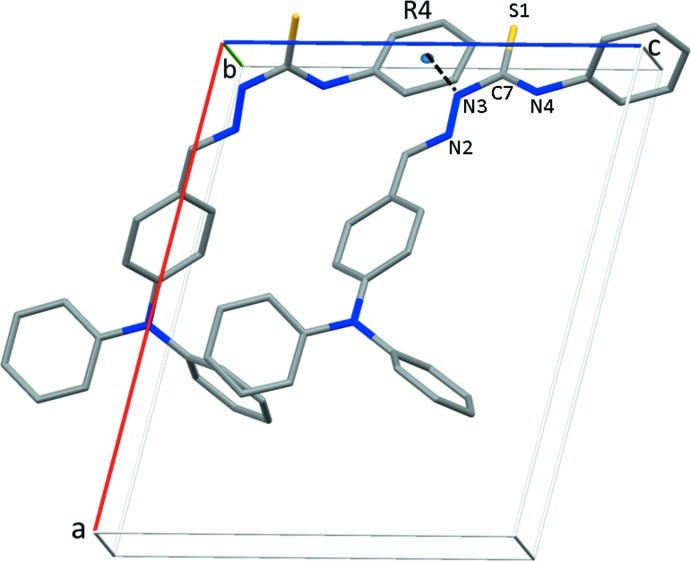
Representation of the weak π–π stacking interaction between phenyl ring (R4) and the thiosemicarbazone deslocalized system.

## supplementary crystallographic information

### Comment

Thiosemicarbazones are a broad class of biologically active organic compounds with antibacterial (Gupta *et al.*,2007) and antitumoral (Odenike *et al.*, 2008) properties. According to recent studies, apolar groups in thiosemicarbazone compounds enhanced in some cases the biological activity (Lee *et al.*, 2010). Following this work line, we have synthesized and crystallized a new thiosemicarbazone (Fig. 1), which is composed of three main parts: triphenylamine group (R1R2R3 *lipophylic domain*) conected to phenyl ring (R4 -*liphophilic group*) by thiosemicarbazone moiety (*H-bonding domain* an and *electron-donor group*) (Fig. 2).

Molecule is the *trans isomer* with respect to the C═N double bond. The values of distances N(2)–N(3) length (1.372 (2) Å) and the dihedral angle C(8)═N(2)—N(3)—C(7) (175.71 (2)°) are similar to those found in CSD (Allen, 2002) for thiosemicarbazone systems [selected 371 hits, distance mean N—N is 1.374 Å and dihedral angle mean is 178.21°]. The N atom in the triaphenylamine is *sp2* and the three benzene rings [(R1(C21–C26), R2(C15–C20 ), R3(C9–C14)] are twisted with respect to one another, with followed dihedral angle between rings [R1R2 = 66.82 (7)° R2R3 = 61.05 (7)° R1R3 = 71.07 (7) °].

Molecular crystals are dominated by strong hydrogen bonds interactions through thiosemicarbazone moiety, forming a centrosymmetry *synthon* through N(3)—H(27)···S(1) hydrogen bond, this interactions form a R^2^_2_(8) graph set (Bernstein *et al.*, 1995). Additional intramolecular hydrogen N(4)—H(28)···N(2) helps to stabilize the molecular conformation (Fig. 3a and 3b).

Taking into account geometrical values calculated with *Platon program* is not feasible the existence π–π interactions involved triaphenylamine group. However, we observed a weak π–π stacking interaction between phenyl ring (R4) and the thiosemicarbazone deslocalized system (C═N—NH—C═S—NH) with distance to N3 (3.834 (3) Å) and dihedral angle (4.68 (2)°) shown in Fig. 4.

### Experimental

A solution of 4-(diphenylamino)benzaldehyde (2.7333 g, 0.01 mol) and 4-phenylthiosemicarbazide (1.6723 g, 0.01 mol) in absolute ethanol (70 ml) was refluxed for 4 h in the presence of *p*-toluenesulfonic acid as catalyst, with continuous stirring. On cooling to room temperature the precipitate was filtered off, washed with copious cold ethanol and dried in air. Yellow single crystals of compound (I) were obtained after recrystallization from a solution in ethanol after 2 d.

### Refinement

All H atoms located at the difference Fourier maps and isotropically refined. At the end of the refinement the highest peak in the electron density was 0.201 eÅ ^-3^, while the deepest hole was -0.291 eÅ ^-3^.

### Crystal data

**Table:**

C_26_H_22_N_4_S	*F*(000) = 888
*M**_r_* = 422.55	*D*_x_ = 1.234 Mg m^−^^3^
Monoclinic, *P*2_1_/*c*	Cu *K*α radiation, λ = 1.54180 Å
Hall symbol: -P 2ybc	Cell parameters from 8693 reflections
*a* = 13.6069 (3) Å	θ = 2.9–75.3°
*b* = 15.2763 (3) Å	µ = 1.41 mm^−^^1^
*c* = 11.2778 (2) Å	*T* = 285 K
β = 104.094 (2)°	Plate, dark yellow
*V* = 2273.67 (8) Å^3^	0.17 × 0.09 × 0.05 mm
*Z* = 4	

### Data collection

**Table:**

Oxford Xcalibur diffractometer with Onyx Nova detector	4623 independent reflections
Radiation source: Nova (Cu) X-ray Source	3535 reflections with *I* > 2σ(*I*)
Mirror monochromator	*R*_int_ = 0.067
Detector resolution: 8.2640 pixels mm^-1^	θ_max_ = 75.5°, θ_min_ = 3.4°
ω scans	*h* = −16→16
Absorption correction: multi-scan (*CrysAlis PRO*; Oxford Diffraction, 2010)	*k* = −17→18
*T*_min_ = 0.726, *T*_max_ = 1.000	*l* = −11→14
26370 measured reflections	

### Refinement

**Table:**

Refinement on *F*^2^	Secondary atom site location: difference Fourier map
Least-squares matrix: full	Hydrogen site location: difference Fourier map
*R*[*F*^2^ > 2σ(*F*^2^)] = 0.049	All H-atom parameters refined
*wR*(*F*^2^) = 0.149	*w* = 1/[σ^2^(*F*_o_^2^) + (0.0766*P*)^2^ + 0.2813*P*] where *P* = (*F*_o_^2^ + 2*F*_c_^2^)/3
*S* = 1.07	(Δ/σ)_max_ < 0.001
4623 reflections	Δρ_max_ = 0.20 e Å^−^^3^
369 parameters	Δρ_min_ = −0.29 e Å^−^^3^
0 restraints	Extinction correction: *SHELXL97* (Sheldrick, 2008), Fc^*^=kFc[1+0.001xFc^2^λ^3^/sin(2θ)]^-1/4^
Primary atom site location: structure-invariant direct methods	Extinction coefficient: 0.0012 (3)

### Special details

**Table:**

Experimental. Absorption correction: CrysAlisPro, Oxford Diffraction Ltd., Version 1.171.34.36 (release 02-08-2010 CrysAlis171 .NET) (compiled Aug 2 2010,13:00:58) Empirical absorption correction using spherical harmonics, implemented in SCALE3 ABSPACK scaling algorithm.
Geometry. All esds (except the esd in the dihedral angle between two l.s. planes) are estimated using the full covariance matrix. The cell esds are taken into account individually in the estimation of esds in distances, angles and torsion angles; correlations between esds in cell parameters are only used when they are defined by crystal symmetry. An approximate (isotropic) treatment of cell esds is used for estimating esds involving l.s. planes.
Refinement. Refinement of F^2^ against ALL reflections. The weighted R-factor wR and goodness of fit S are based on F^2^, conventional R-factors R are based on F, with F set to zero for negative F^2^. The threshold expression of F^2^ > 2sigma(F^2^) is used only for calculating R-factors(gt) etc. and is not relevant to the choice of reflections for refinement. R-factors based on F^2^ are statistically about twice as large as those based on F, and R-factors based on ALL data will be even larger.

### Fractional atomic coordinates and isotropic or equivalent isotropic displacement parameters (Å^2^)

**Table:**

	*x*	*y*	*z*	*U*_iso_*/*U*_eq_	
S1	0.94600 (4)	0.03619 (4)	0.15627 (5)	0.0689 (2)	
N2	1.16947 (11)	0.16240 (10)	0.07520 (14)	0.0504 (4)	
N3	1.08689 (11)	0.10941 (11)	0.06823 (15)	0.0522 (4)	
N4	1.08031 (14)	0.15773 (11)	0.25732 (15)	0.0591 (4)	
N1	1.56053 (12)	0.35772 (12)	−0.02307 (15)	0.0597 (4)	
C8	1.21068 (14)	0.15830 (13)	−0.01584 (17)	0.0520 (4)	
C14	1.33120 (14)	0.27924 (13)	0.06329 (17)	0.0527 (4)	
C9	1.29891 (13)	0.21151 (12)	−0.01924 (16)	0.0490 (4)	
C7	1.04293 (14)	0.10515 (12)	0.16271 (16)	0.0510 (4)	
C12	1.47208 (13)	0.30889 (12)	−0.02447 (17)	0.0508 (4)	
C13	1.41611 (15)	0.32736 (13)	0.06068 (18)	0.0534 (4)	
C15	1.58656 (15)	0.38294 (13)	−0.13237 (19)	0.0554 (5)	
C11	1.43998 (16)	0.24198 (14)	−0.10803 (19)	0.0611 (5)	
C10	1.35392 (16)	0.19418 (14)	−0.10567 (18)	0.0585 (5)	
C6	1.04960 (16)	0.15504 (13)	0.37043 (18)	0.0573 (5)	
C21	1.62500 (14)	0.38028 (13)	0.09250 (18)	0.0559 (5)	
C20	1.68751 (18)	0.39145 (16)	−0.1348 (3)	0.0717 (6)	
C5	0.97506 (19)	0.20986 (19)	0.3890 (2)	0.0749 (6)	
C16	1.5132 (2)	0.40044 (16)	−0.2379 (2)	0.0706 (6)	
C22	1.66006 (18)	0.46481 (15)	0.1163 (2)	0.0680 (6)	
C3	0.9924 (3)	0.1471 (2)	0.5872 (3)	0.0880 (8)	
C1	1.0965 (2)	0.09751 (18)	0.4592 (2)	0.0832 (7)	
C19	1.7123 (3)	0.4176 (2)	−0.2411 (4)	0.0966 (10)	
C23	1.7227 (2)	0.4862 (2)	0.2285 (3)	0.0841 (7)	
C17	1.5399 (3)	0.4254 (2)	−0.3436 (3)	0.0895 (8)	
C18	1.6401 (3)	0.4340 (2)	−0.3447 (4)	0.1005 (10)	
C26	1.6521 (2)	0.31817 (18)	0.1832 (2)	0.0818 (7)	
C2	1.0684 (3)	0.0947 (2)	0.5689 (3)	0.1006 (10)	
C4	0.9469 (2)	0.2052 (2)	0.5001 (3)	0.0877 (8)	
C24	1.7493 (2)	0.4241 (2)	0.3180 (3)	0.0984 (9)	
C25	1.7131 (3)	0.3415 (2)	0.2959 (3)	0.1070 (11)	
H8	1.1832 (15)	0.1157 (13)	−0.0856 (18)	0.052 (5)*	
H13	1.4370 (18)	0.3751 (16)	0.116 (2)	0.069 (6)*	
H10	1.3326 (17)	0.1481 (15)	−0.163 (2)	0.064 (6)*	
H27	1.0675 (17)	0.0693 (16)	−0.001 (2)	0.069 (6)*	
H14	1.2919 (17)	0.2952 (15)	0.123 (2)	0.067 (6)*	
H28	1.131 (2)	0.1912 (18)	0.249 (2)	0.076 (7)*	
H11	1.4793 (19)	0.2228 (16)	−0.164 (2)	0.076 (7)*	
H22	1.639 (2)	0.509 (2)	0.055 (3)	0.088 (8)*	
H5	0.945 (2)	0.249 (2)	0.327 (3)	0.094 (9)*	
H20	1.737 (2)	0.3775 (17)	−0.064 (2)	0.076 (7)*	
H16	1.446 (2)	0.3934 (18)	−0.237 (2)	0.085 (8)*	
H1	1.157 (3)	0.057 (2)	0.454 (3)	0.116 (10)*	
H26	1.631 (2)	0.259 (2)	0.162 (3)	0.099 (9)*	
H23	1.743 (2)	0.545 (2)	0.249 (3)	0.100 (9)*	
H4	0.893 (3)	0.242 (3)	0.507 (3)	0.127 (12)*	
H3	0.970 (3)	0.139 (2)	0.657 (3)	0.110 (10)*	
H17	1.486 (2)	0.4377 (19)	−0.416 (3)	0.095 (9)*	
H25	1.729 (2)	0.295 (2)	0.356 (3)	0.114 (10)*	
H18	1.650 (3)	0.453 (2)	−0.425 (3)	0.123 (11)*	
H24	1.793 (3)	0.435 (2)	0.402 (3)	0.117 (10)*	
H2	1.094 (3)	0.047 (3)	0.627 (4)	0.156 (15)*	
H19	1.776 (3)	0.418 (2)	−0.242 (3)	0.115 (11)*	

### Atomic displacement parameters (Å^2^)

**Table:**

	*U*^11^	*U*^22^	*U*^33^	*U*^12^	*U*^13^	*U*^23^
S1	0.0620 (3)	0.0902 (4)	0.0645 (3)	−0.0305 (3)	0.0346 (3)	−0.0166 (2)
N2	0.0435 (8)	0.0544 (9)	0.0571 (9)	−0.0051 (6)	0.0197 (7)	0.0061 (6)
N3	0.0470 (8)	0.0611 (9)	0.0538 (9)	−0.0103 (6)	0.0227 (7)	0.0001 (7)
N4	0.0621 (10)	0.0633 (10)	0.0595 (10)	−0.0172 (8)	0.0291 (8)	−0.0071 (7)
N1	0.0513 (9)	0.0729 (11)	0.0580 (10)	−0.0204 (7)	0.0194 (7)	0.0007 (7)
C8	0.0469 (10)	0.0628 (11)	0.0496 (10)	−0.0083 (8)	0.0181 (8)	0.0027 (8)
C14	0.0495 (10)	0.0655 (11)	0.0478 (10)	−0.0069 (8)	0.0208 (8)	−0.0009 (8)
C9	0.0435 (9)	0.0603 (10)	0.0457 (9)	−0.0073 (7)	0.0158 (7)	0.0046 (7)
C7	0.0456 (10)	0.0591 (11)	0.0518 (10)	−0.0002 (7)	0.0187 (8)	0.0028 (7)
C12	0.0446 (9)	0.0591 (10)	0.0511 (10)	−0.0118 (7)	0.0164 (8)	0.0016 (7)
C13	0.0527 (11)	0.0601 (11)	0.0504 (10)	−0.0097 (8)	0.0180 (8)	−0.0041 (8)
C15	0.0517 (10)	0.0556 (10)	0.0659 (12)	−0.0092 (8)	0.0274 (9)	0.0009 (8)
C11	0.0612 (12)	0.0715 (13)	0.0593 (12)	−0.0200 (9)	0.0315 (10)	−0.0112 (9)
C10	0.0605 (12)	0.0674 (12)	0.0533 (11)	−0.0206 (9)	0.0244 (9)	−0.0099 (9)
C6	0.0601 (11)	0.0618 (11)	0.0555 (11)	−0.0156 (8)	0.0248 (9)	−0.0101 (8)
C21	0.0446 (10)	0.0610 (11)	0.0626 (11)	−0.0097 (8)	0.0141 (8)	0.0007 (8)
C20	0.0556 (13)	0.0799 (15)	0.0902 (17)	−0.0125 (10)	0.0380 (13)	−0.0069 (12)
C5	0.0682 (14)	0.0901 (17)	0.0703 (15)	0.0024 (12)	0.0241 (12)	−0.0077 (12)
C16	0.0648 (14)	0.0816 (15)	0.0711 (14)	−0.0047 (11)	0.0274 (11)	0.0119 (11)
C22	0.0681 (14)	0.0619 (13)	0.0732 (14)	−0.0122 (10)	0.0160 (11)	−0.0012 (10)
C3	0.109 (2)	0.100 (2)	0.0674 (16)	−0.0263 (16)	0.0463 (16)	−0.0164 (14)
C1	0.109 (2)	0.0796 (16)	0.0722 (15)	0.0111 (14)	0.0446 (14)	0.0038 (11)
C19	0.088 (2)	0.100 (2)	0.125 (3)	−0.0330 (16)	0.071 (2)	−0.0205 (18)
C23	0.0795 (17)	0.0834 (18)	0.0887 (18)	−0.0274 (13)	0.0189 (13)	−0.0199 (14)
C17	0.111 (2)	0.0909 (19)	0.0725 (17)	−0.0087 (15)	0.0344 (16)	0.0171 (13)
C18	0.133 (3)	0.097 (2)	0.094 (2)	−0.0332 (18)	0.070 (2)	0.0010 (16)
C26	0.0736 (15)	0.0710 (15)	0.0876 (17)	−0.0168 (12)	−0.0056 (12)	0.0146 (12)
C2	0.144 (3)	0.097 (2)	0.0718 (17)	0.0073 (19)	0.0487 (18)	0.0130 (14)
C4	0.0747 (17)	0.111 (2)	0.0872 (19)	−0.0094 (15)	0.0392 (15)	−0.0299 (16)
C24	0.0808 (18)	0.124 (3)	0.0789 (18)	−0.0342 (17)	−0.0022 (14)	−0.0033 (16)
C25	0.097 (2)	0.111 (2)	0.091 (2)	−0.0268 (17)	−0.0221 (16)	0.0267 (17)

### Geometric parameters (Å, º)

**Table:**

S1—C7	1.6757 (19)	C21—C26	1.378 (3)
N2—C8	1.286 (2)	C21—C22	1.380 (3)
N2—N3	1.372 (2)	C20—C19	1.380 (4)
N3—C7	1.345 (2)	C20—H20	0.94 (3)
N3—N2	1.372 (2)	C5—C4	1.399 (4)
N3—H27	0.98 (2)	C5—H5	0.94 (3)
N4—C7	1.333 (2)	C16—C17	1.382 (3)
N4—C6	1.436 (2)	C16—H16	0.92 (3)
N4—H28	0.88 (3)	C22—C23	1.381 (4)
N1—C12	1.413 (2)	C22—H22	0.96 (3)
N1—C15	1.416 (2)	C3—C4	1.357 (5)
N1—C21	1.425 (3)	C3—C2	1.362 (5)
C8—N2	1.286 (2)	C3—H3	0.92 (4)
C8—C9	1.458 (2)	C1—C2	1.381 (4)
C8—H8	1.02 (2)	C1—H1	1.04 (4)
C14—C13	1.376 (3)	C19—C18	1.354 (5)
C14—C9	1.390 (3)	C19—H19	0.87 (4)
C14—H14	0.98 (2)	C23—C24	1.368 (4)
C9—C10	1.391 (3)	C23—H23	0.95 (3)
C12—C11	1.387 (3)	C17—C18	1.373 (5)
C12—C13	1.392 (3)	C17—H17	0.98 (3)
C13—H13	0.96 (2)	C18—H18	0.99 (4)
C15—C16	1.381 (3)	C26—C25	1.385 (4)
C15—C20	1.387 (3)	C26—H26	0.97 (3)
C11—C10	1.386 (3)	C2—H2	0.99 (5)
C11—H11	0.97 (3)	C4—H4	0.94 (4)
C10—H10	0.95 (2)	C24—C25	1.356 (5)
C6—C1	1.368 (4)	C24—H24	1.01 (3)
C6—C5	1.370 (3)	C25—H25	0.97 (4)
			
C8—N2—N3	115.86 (16)	C19—C20—H20	121.7 (17)
C7—N3—N2	119.97 (16)	C15—C20—H20	118.5 (17)
C7—N3—H27	121.1 (14)	C6—C5—C4	118.7 (3)
N2—N3—H27	118.4 (14)	C6—C5—H5	118.7 (18)
C7—N4—C6	123.82 (16)	C4—C5—H5	122.6 (18)
C7—N4—H28	114.7 (17)	C15—C16—C17	120.7 (3)
C6—N4—H28	121.1 (17)	C15—C16—H16	118.7 (17)
C12—N1—C15	121.79 (16)	C17—C16—H16	120.6 (17)
C12—N1—C21	118.09 (15)	C21—C22—C23	120.4 (2)
C15—N1—C21	120.11 (15)	C21—C22—H22	118.8 (17)
N2—C8—C9	121.06 (17)	C23—C22—H22	120.8 (17)
N2—C8—H8	119.9 (11)	C4—C3—C2	120.3 (3)
C9—C8—H8	119.0 (11)	C4—C3—H3	121 (2)
C13—C14—C9	120.91 (18)	C2—C3—H3	118 (2)
C13—C14—H14	118.7 (13)	C6—C1—C2	119.6 (3)
C9—C14—H14	120.3 (13)	C6—C1—H1	125.0 (19)
C14—C9—C10	118.33 (16)	C2—C1—H1	115.3 (19)
C14—C9—C8	121.71 (17)	C18—C19—C20	121.6 (3)
C10—C9—C8	119.95 (17)	C18—C19—H19	120 (2)
N4—C7—N3	116.64 (16)	C20—C19—H19	118 (2)
N4—C7—S1	123.79 (14)	C24—C23—C22	120.4 (3)
N3—C7—S1	119.57 (14)	C24—C23—H23	118.0 (18)
C11—C12—C13	118.91 (16)	C22—C23—H23	121.4 (18)
C11—C12—N1	121.55 (17)	C18—C17—C16	120.3 (3)
C13—C12—N1	119.53 (17)	C18—C17—H17	121.6 (18)
C14—C13—C12	120.66 (18)	C16—C17—H17	118.1 (18)
C14—C13—H13	120.6 (14)	C19—C18—C17	119.2 (3)
C12—C13—H13	118.7 (14)	C19—C18—H18	128 (2)
C16—C15—C20	118.5 (2)	C17—C18—H18	113 (2)
C16—C15—N1	121.41 (18)	C21—C26—C25	119.8 (3)
C20—C15—N1	120.1 (2)	C21—C26—H26	117.1 (18)
C10—C11—C12	120.20 (19)	C25—C26—H26	122.9 (18)
C10—C11—H11	117.2 (14)	C3—C2—C1	120.2 (3)
C12—C11—H11	122.3 (14)	C3—C2—H2	120 (3)
C11—C10—C9	120.97 (18)	C1—C2—H2	119 (3)
C11—C10—H10	119.6 (14)	C3—C4—C5	120.4 (3)
C9—C10—H10	119.4 (14)	C3—C4—H4	124 (2)
C1—C6—C5	120.8 (2)	C5—C4—H4	116 (2)
C1—C6—N4	118.9 (2)	C25—C24—C23	119.4 (3)
C5—C6—N4	120.3 (2)	C25—C24—H24	116 (2)
C26—C21—C22	118.8 (2)	C23—C24—H24	125 (2)
C26—C21—N1	120.42 (19)	C24—C25—C26	121.2 (3)
C22—C21—N1	120.73 (19)	C24—C25—H25	123 (2)
C19—C20—C15	119.8 (3)	C26—C25—H25	116 (2)
			
C8—N2—N3—C7	175.71 (17)	C7—N4—C6—C1	85.9 (3)
N3—N2—C8—C9	179.95 (16)	C7—N4—C6—C5	−94.6 (3)
C13—C14—C9—C10	−0.8 (3)	C12—N1—C21—C26	46.8 (3)
C13—C14—C9—C8	178.35 (18)	C15—N1—C21—C26	−132.2 (2)
N2—C8—C9—C14	−11.5 (3)	C12—N1—C21—C22	−132.1 (2)
N2—C8—C9—C14	−11.5 (3)	C15—N1—C21—C22	48.9 (3)
N2—C8—C9—C10	167.58 (19)	C16—C15—C20—C19	0.5 (4)
N2—C8—C9—C10	167.58 (19)	N1—C15—C20—C19	−178.8 (2)
C6—N4—C7—N3	−173.22 (18)	C1—C6—C5—C4	−0.7 (4)
C6—N4—C7—S1	7.0 (3)	N4—C6—C5—C4	179.8 (2)
N2—N3—C7—N4	3.5 (3)	C20—C15—C16—C17	0.3 (4)
N2—N3—C7—N4	3.5 (3)	N1—C15—C16—C17	179.7 (2)
N2—N3—C7—S1	−176.74 (13)	C26—C21—C22—C23	1.1 (4)
N2—N3—C7—S1	−176.74 (13)	N1—C21—C22—C23	−179.9 (2)
C15—N1—C12—C11	39.0 (3)	C5—C6—C1—C2	−0.1 (4)
C21—N1—C12—C11	−139.9 (2)	N4—C6—C1—C2	179.4 (3)
C15—N1—C12—C13	−141.7 (2)	C15—C20—C19—C18	−1.1 (4)
C21—N1—C12—C13	39.3 (3)	C21—C22—C23—C24	−1.0 (4)
C9—C14—C13—C12	−0.4 (3)	C15—C16—C17—C18	−0.7 (4)
C11—C12—C13—C14	1.0 (3)	C20—C19—C18—C17	0.8 (5)
N1—C12—C13—C14	−178.29 (18)	C16—C17—C18—C19	0.1 (5)
C12—N1—C15—C16	31.7 (3)	C22—C21—C26—C25	0.3 (4)
C21—N1—C15—C16	−149.4 (2)	N1—C21—C26—C25	−178.6 (3)
C12—N1—C15—C20	−149.0 (2)	C4—C3—C2—C1	−3.0 (5)
C21—N1—C15—C20	30.0 (3)	C6—C1—C2—C3	1.9 (5)
C13—C12—C11—C10	−0.4 (3)	C2—C3—C4—C5	2.2 (5)
N1—C12—C11—C10	178.82 (19)	C6—C5—C4—C3	−0.3 (4)
C12—C11—C10—C9	−0.7 (3)	C22—C23—C24—C25	−0.5 (5)
C14—C9—C10—C11	1.3 (3)	C23—C24—C25—C26	2.0 (6)
C8—C9—C10—C11	−177.8 (2)	C21—C26—C25—C24	−2.0 (6)

### Hydrogen-bond geometry (Å, º)

**Table:**

*D*—H···*A*	*D*—H	H···*A*	*D*···*A*	*D*—H···*A*
N4—H28···N2	0.88 (3)	2.19 (3)	2.629 (2)	110 (2)
N3—H27···S1^i^	0.98 (2)	2.36 (3)	3.318 (2)	169 (2)

Symmetry code: (i) −*x*+2, −*y*, −*z*.
